# Sleep Duration and Behaviours: A Descriptive Analysis of a Cohort of Dogs up to 12 Months of Age

**DOI:** 10.3390/ani10071172

**Published:** 2020-07-10

**Authors:** Rachel Kinsman, Sara Owczarczak-Garstecka, Rachel Casey, Toby Knowles, Séverine Tasker, Joshua Woodward, Rosa Da Costa, Jane Murray

**Affiliations:** 1 Dogs Trust, London EC1V 7RQ, UK; rachel.casey@dogstrust.org.uk (R.C.); joshua.woodward@dogstrust.org.uk (J.W.); rosa.dacosta@dogstrust.org.uk (R.D.C.); jane.murray@dogstrust.org.uk (J.M.); 2 Bristol Veterinary School, University of Bristol, Bristol BA6 8DD, UK; toby.knowles@bristol.ac.uk (T.K.); s.tasker@bristol.ac.uk (S.T.); 3 Linnaeus Group, Shirley, West Midlands B90 4BN, UK

**Keywords:** dogs, sleep, sleep duration, sleep behaviours, sleep habits, sleeping positions, co-sleeping, human–animal interaction, companion animals

## Abstract

**Simple Summary:**

Sleep in dogs is a rarely studied but important behaviour. Changes in the pattern and duration of a dog’s sleep can reflect a dog’s wakeful experiences and how comfortable they are in their own environment. Little is known about normal sleep behaviours in dogs under 12 months of age. This study aimed to describe patterns of sleep and sleep-related behaviours (such as where the dog slept, how the dog was settled to sleep, sleep positions, and snoring) based on reports from owners of dogs aged 16 weeks and 12 months. For the statistical analysis, only dogs with data regarding sleep duration at both timepoints were used. Dogs aged 16 weeks slept for significantly longer during the day and in total over a 24 h period, but for less time during the night than dogs at 12 months. At both timepoints, owners most commonly settled dogs to sleep by leaving the dog in a room/area without human company. However, of dogs that did have access to people during the night, more than 86% chose to be around people. Puppies aged 16 weeks were most commonly reported to sleep in a kennel/crate, but dogs aged 12 months most often slept in a dog bed. More research is needed to better understand how the sleep duration and behaviours of dogs change as they age, and how sleep can affect dog health and wellbeing.

**Abstract:**

Sleep is a vital behaviour that can reflect an animal’s adaptation to the environment and their welfare. However, a better understanding of normal age-specific sleep patterns is crucial. This study aims to provide population norms and descriptions of sleep-related behaviours for 16-week-old puppies and 12-month-old dogs living in domestic environments. Participants recruited to a longitudinal study answered questions relating to their dogs’ sleep behaviours in surveys issued to them when their dogs reached 16 weeks (*n* = 2332) and 12 months of age (*n* = 1091). For the statistical analysis, subpopulations of dogs with data regarding sleep duration at both timepoints were used. Owners of 16-week-old puppies perceived their dogs to sleep longer during the day and over a 24 h period, but for less time during the night than owners of 12-month-old dogs. At both timepoints, dogs were most commonly settled to sleep by being left in a room/area without human company. However, of dogs that had access to people overnight, 86.7% and 86.8% chose to be around people at 16 weeks and 12 months of age, respectively. The most common sleeping place was in a kennel/crate at 16 weeks (49.1%), and a dog bed at 12 months (31.7%). Future research within this longitudinal study will investigate how sleep duration and behaviours change with age and impact on a dog’s health and behaviour.

## 1. Introduction

Sleep, defined as a reversible “state of immobility with greatly reduced responsiveness” [1] (p. 1264) is a vital behaviour in all mammals, including dogs. The primary function of sleep is often debated and may vary between species [1]. However, learning and memory consolidation, as well as maintenance of the immune system [2] appear to be consistently important. In addition, changes in animal sleep behaviour (e.g., increased vigilance during a sleep period resulting in a reduced sleep duration) may be indicative of animals’ ability to cope with their environment or changes in environment [3,4,5]. To use sleep behaviours as a part of a dog’s welfare assessment, age-specific population norms and descriptions of sleep behaviours are needed. However, to the best knowledge of the authors, such norms, and descriptions for dogs under the age of 12 months, do not exist. This paper aims to address this gap in knowledge.

Sleep behaviour in individual dogs may be shaped by a multitude of factors: sleep structure (i.e., latency to the first sleep bout, sequence of Rapid Eye Movement (REM) and non-REM sleep, number of REM and non-REM phases and structure of brainwaves associated with REM, non-REM and drowsiness) and duration change in response to events during wakefulness [6]. For instance, learning a novel task influences both REM and non-REM sleep structure in dogs [7,8]. Changes in sleep structure are also predictive of dog performance on a task afterwards, illustrating the relationship between sleep and memory consolidation [7,8]. Sleep architecture and duration are also altered in response to emotionally-valenced events. Dogs who experienced interactions likely to induce a negative affect have a shorter sleep latency and show redistribution of sleep stages compared to dogs that experienced interactions likely to induce a positive affect [9]. Sleep structure and duration are also modulated by a dog’s personality and behaviour [9]. In addition, dogs’ activity levels, variation from routine, social interactions with other dogs and humans, and other emotional experiences [10,11,12] can also affect sleep. Dogs that are physically active during the day show a different sleep structure compared to dogs that are inactive [13]. Moreover, diet and feeding frequency [14], environmental enrichment and changes in housing have been demonstrated to impact on dogs’ sleep [13,14,15]. Human–dog co-sleeping (i.e., when a dog and a person sleep in the same bedroom) affects human sleep patterns [16,17]. It is plausible that the opposite is also true, and that dogs’ sleep is also influenced by humans’ sleep. Finally, dogs’ sleep may be related to breed. For example, previous research suggests that anatomical abnormalities in brachycephalic breeds lead to breathing difficulties during sleep [18]. However, links between breed and sleep structure or duration have not previously been explored. It has also been hypothesised that sleep in animals is correlated with their body and brain mass, with larger animals sleeping less than smaller animals [19], but it is unclear whether dogs of different sizes follow a different sleep pattern [20].

Previous research shows that dogs follow a diurnal circadian rhythm (i.e., are active during the daylight and sleep primarily during the nighttime) [15]. Past studies have reported that, on average, dogs older than 1.5 years of age sleep between 60 and 80% of the night (typically defined as time between 8 p.m. and 8 a.m.) [3,15,21] and 3 and 28% [3,21] of daytime (i.e., time between 8 a.m. and 8 p.m.) [14]. Other sources suggest that over a 24 h cycle, dogs sleep on average 10.1 h [19]. Sleep pattern is linked with age of the dog [14,21,22]. Older dogs sleep substantially more during the day and nighttime and have shorter but more frequent sleep bouts during the day [14,22]. Patterns of sleep in dogs under 1.5 years of age are largely unknown.

The relationship between sleep and animal welfare is circular: conditions affecting welfare may impact sleep quality and a deterioration in sleep quality may have a negative effect on animal welfare. Sleep behaviour can reflect animal adaptation to the environment, and in this way, can be used as a proxy indicator of animal welfare [3,4,5]. Changes in sleep and resting behaviour, such as increased inactivity while awake, are linked with a compromised welfare in cats [23], rats [24] and mink [25]. Lack of sleep and sleep restrictions are a source of distress and are associated with poor emotional regulation, psychomotor and sensorimotor impairments, aggression, anxiety and poor ability to cope with stressful stimuli [26,27,28], potentially further aggravating the impact of loss of sleep on welfare. Simultaneously, sleep and resting behaviour can indicate ability to cope with the environment and positive welfare. For example, shelter dogs that are better able to cope with the rescue environment rest and sleep more during the day [3]. In addition, sleeping postures and where a dog sleeps can indicate an animal’s welfare. For instance, anxiety may promote sleeping in positions that enable quick escape [25] as well as more interrupted sleep and resting bouts, as animals are more likely to remain vigilant [29]. As puppies are typically acquired at the age of 8 weeks [30], sleep behaviour in the first year of life can also offer insight into the puppy’s adaptation to the new home environment and routine.

In order to use sleep behaviour as an indicator of welfare in dogs, a better understanding of normal age-specific sleep patterns and behaviours (such as sleeping positions and locations) is crucial. Several methods of recording sleep patterns in dogs exist. In particular, polysomnography has been highlighted as having good ecological validity (i.e., dogs can be observed in their natural environments) and specificity (i.e., it has been validated against other methods) [12,20]. However, polysomnography requires extensive equipment, which may limit the number of the observed dogs and may not be appropriate for puppies and young dogs.

In clinical contexts, sleep patterns are most likely to be gauged from dog owners’ reports. Therefore, a better understanding of sleep pattern in dogs based on this observational method is still needed, even if owner’s reports may be inaccurate. Previous research frequently relied on small sample sizes (e.g., *n* = 1; [27]) [15] and brief periods of observations aimed to explore the sleep architecture and brain activity rather than the overall sleep pattern (e.g., *n* = 3 h) [12]. Some studies were also conducted in experimental conditions where dogs were kept in small crates, limiting the ecological and external validity of the reported findings [22]. Exploration of sleep based on a large sample of dogs within their own home environment over a period of time is, therefore, needed to establish population norms. The choices owners make for their dog at specific ages (such as how their dog is settled to sleep, the use of a crate/kennel while toilet training, and the location of their dog’s bed) could impact on the dog’s behaviour and future behaviours (including sleep behaviour), and also require investigation.

Consequently, this study aimed to summarise owner-reported sleep duration, characteristics, and presence of a range of sleep behaviours in a large sample of companion dogs at ages 16 weeks and 12 months. Data reported in this study could be used as a reference when exploring sleep as a proxy indicator of welfare of dogs. Future research will investigate the impact of sleep behaviour on specific health and behaviour problems occurring within the ‘Generation Pup’ cohort.

## 2. Materials and Methods

### 2.1. Study Design and Participants

Data for this study were collected as part of ‘Generation Pup’—a longitudinal study of canine health, behaviour and welfare. The inclusion criteria for ‘Generation Pup’ were that the participant must: (1) be resident in the United Kingdom (UK) or the Republic of Ireland (ROI), (2) be at least 16 years of age, and (3) own a puppy (any breed or cross-breed) under 16 weeks of age at time of registration. ‘Generation Pup’ intends to recruit 10,000 puppies, so the data utilized in this study were extracted whilst recruitment was still in progress.

This analysis uses data from 4002 puppies that were recruited between May 2016 and March 2020. To ensure recruitment of a diverse group of dog owners, a variety of recruitment methods were used including advertising through puppy training classes, veterinary practices, dog breeders, social media and publications (e.g., Veterinary Record and Dogs Trust WAG magazine).

### 2.2. Data Collection

For this study, data were obtained from three online or postal self-administered surveys. The first survey was completed upon registration to the project (age of the puppy varied from birth to 16 weeks of age), and two that were issued to owners when their dogs were 16 weeks and 12 months of age. The surveys contained primarily closed questions with multiple-choice answers, and free-text responses were recoded where required. Appendix A shows the sleep-related questions from the ‘Generation Pup’ 16 week survey and 12 month survey. Prior to analysis, all data were pseudonymised.

The study had ethical approval from the University of Bristol Animal Welfare Ethical Research Board (UIN/18/052), the Clinical Research Ethical Review Board at the Royal Veterinary College-URN 2017 1658-3, the Social Science Ethical Review Board at the Royal Veterinary College-URN SR2017-1116, and Dogs Trust Ethical Review Board–ERB009.

### 2.3. Description of Variables

Owners were asked to approximate the minimum and maximum numbers of hours their dogs slept in an average 24 h period during the daytime and during the nighttime at both timepoints. Owners were not asked to remain awake to monitor dogs’ sleep, and therefore their approximations are likely to be based on observing the time the dog went to sleep and woke up as well as any instances of dog being awake during the nighttime. The duration of “daytime” and “nighttime” was not specified and was left to owners’ interpretation. For dogs with both minimum and maximum values for time slept, these values were used to calculate average durations of daytime and nighttime sleep for individual dogs. Average durations of daytime and nighttime sleep for individual dogs were then used to calculate the mean time slept during the daytime and nighttime for the study sample. Where both daytime and nighttime data were available for an individual dog, total sleep time per 24 h period was calculated using the daytime and nighttime averages. The average durations of total sleep for individual dogs were then used to calculate the mean time slept in total for the study sample. Dogs were excluded from the analysis if their owners were unsure how long their dog had slept.

Presence of a range of sleep-related behaviours and characteristics (summarised in Table 1, Table 2 and Table 3) was also recorded. In both the 16 week and 12 month surveys, owners were asked how their dog was settled to sleep at night and what their dog slept on/in at night during the last seven days. Owners were given a pre-defined list of responses for both questions and a free-text “other” option (Table 1). Information regarding access to people during the night and whether the dog typically chose to be close to people at night was also collected (Table 1). In the 12 month survey, owners were also able to select their dog’s usual sleeping positions and report their observations of the dog sleep behaviours from a pre-defined list and/or add an “other” response if needed (Table 2). Based on the number of dogs with data available for the behaviour ‘often snored very loudly’, the percentage of dogs reported exhibiting this behaviour was calculated for the seven breeds most frequently reported to ‘often snore very loudly’ (Table 3).

### 2.4. Study Size

The study size was determined by the number of dogs whose owners had completed the 16 week and 12 month surveys and had answered questions about their dog’s sleep. To remove any effects of clustering at the level of the household, if an owner had registered more than one puppy onto the project, only one of their puppies was randomly selected for inclusion in the dataset. At the time of this analysis, the number of puppies recruited to the ‘Generation Pup’ study was 4002; 138 were excluded, as they lived in a household with another dog registered in the study.

**Figure 1:**
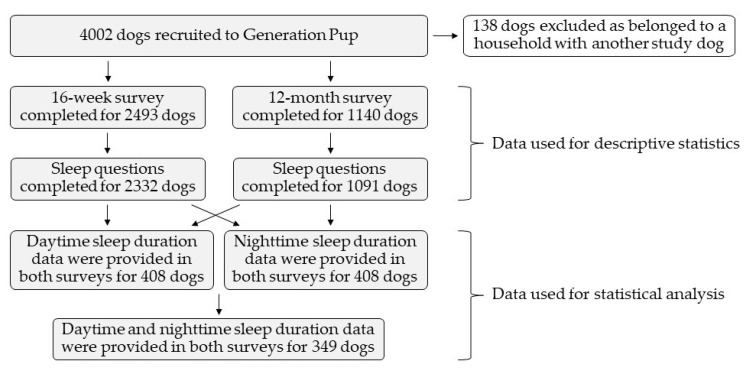
A flowchart detailing the inclusion of dogs from the ‘Generation Pup’ cohort in the descriptive statistics and statistical analysis carried out in this study.

The 16 week survey was completed for 2493 dogs and the sleep questions were answered for 2332 dogs. The 12 month survey was completed for 1140 dogs and responses to sleep questions were provided for 1091 dogs. Throughout, all available data were used for summary statistics. However, for the statistical analysis of average sleep during (1) daytime, (2) nighttime, and (3) in total over a 24 h period, three subpopulations of dogs where owners provided data in both the 16 week and 12 month timepoints were used (Figure 1).

Owner-perceived sleep duration data were provided in both the 16 week and 12 month surveys for 408 dogs, 662 dogs, and 349 dogs for (1) daytime, (2) nighttime, and (3) both nighttime and daytime sleep durations (used to calculate total sleep in a 24 h period), respectively (Figure 1).

### 2.5. Descriptive Statistics

Frequency of sleep-related behaviours and sleep characteristics was summarised.

### 2.6. Statistical Analysis

Duration of daytime, nighttime and total sleep data were explored graphically, and normality was assessed with Shapiro–Wilk tests. (One dog was removed from the 16 week daytime sleep histogram and one dog was removed from the 16 week total sleep histogram due to being outliers, which were likely to be reported in error.)

For the three subpopulations of dogs with data at both timepoints, paired-sample *t*-tests (where the data followed a normal distribution) and Mann–Whitney tests (where the data did not follow a normal distribution) were conducted to compare the duration of sleep during the daytime, nighttime, and in total for dogs aged 16 weeks and 12 months.

## 3. Results

### 3.1. Duration of Owner-Perceived Sleep

**Figure 2:**
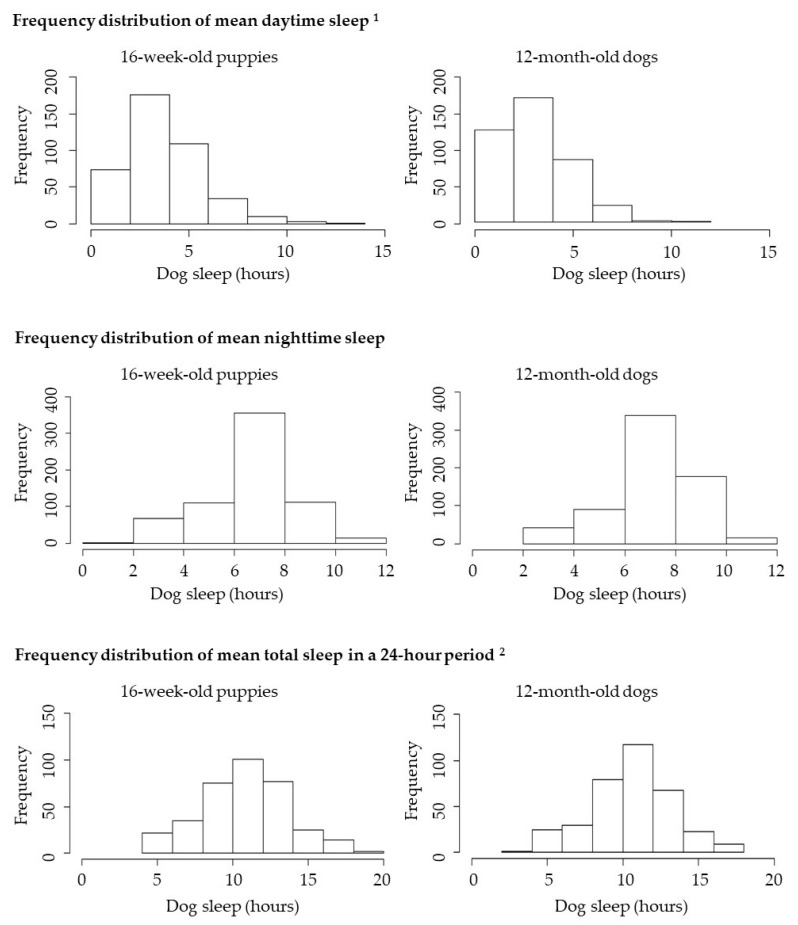
Summary of owner-reported sleep duration at daytime (*n* = 408), nighttime (*n* = 662) and in total over a 24 h period (*n* = 349) for dogs with data reported in both the 16 week and 12 month surveys. ^1^ One dog was removed from the 16 week daytime sleep histogram due to being an outlier. ^2^ One dog was removed from the 16 week total sleep histogram due to being an outlier.

When owners were asked whether they knew approximately how long their 16-week-old puppies slept during the daytime, 58.1% (1354/2332) of owners reported approximate minimum and maximum numbers of hours that their puppies slept. A slightly smaller percentage of owners, 48.6% (530/1091), were able to report this information for their dogs when they reached 12 months of age, and 408 owners reported this information at both timepoints. For the subpopulation of 408 dogs, the duration of daytime sleep at both 16 weeks and 12 months was right skewed (Figure 2). The median time asleep during the day was reported by owners as 3.5 h (IQR = 2.5) and 3.0 h (IQR = 2.5) in the 16 week and 12 month surveys, respectively (Figure 2). The statistical analysis of daytime sleep of the subpopulation of 408 dogs revealed the difference in the duration of daytime sleep between the two timepoints was statistically significant (V = 45480, *p* ≤ 0.001).

When owners were asked whether they knew approximately how long their dog slept during the nighttime, 81.3% (1896/2332) and 75.2% (820/1091) of owners reported both minimum and maximum numbers of hours at the 16 week and 12 month timepoints, respectively. Data were available at both timepoints for 662 dogs. For this subpopulation of 662 dogs, the duration of nighttime sleep at both timepoints was normally distributed (Figure 2). The mean time asleep at night was reported as 7.0 h (SD = 1.7) and 7.3 h (SD = 1.6) at 16 weeks and 12 months, respectively. The mean difference in nighttime sleep between the two timepoints was 0.4 h; t(661) = −5.5, *p* < 0.001 (95% confidence interval −0.5–−0.2).

For the 352 dogs for whom daytime and nighttime sleep was available at both timepoints, the total sleep duration over a 24 h period followed a normal distribution. The mean total hours of sleep in this subpopulation was reported as 11.2 h (SD = 2.9) and 10.8 h (SD = 2.7) at 16 weeks and 12 months, respectively (Figure 2). The mean difference in total time asleep between the two timepoints was 0.3 h; t(351) = 2.4, *p* < 0.05 (95% confidence interval 0.1–0.6). Therefore, while the duration of nighttime sleep was reported to be longer in the 12 month survey than in the 16 week survey, the duration of daytime sleep and total sleep was reported to be longer in the 16 week survey.

### 3.2. Sleep Characteristics and Behaviours

A summary of sleep-related data at 16 weeks and 12 months of age is provided in Table 1. From a pre-defined list, owners were asked to select one response that reflected how they settled their dog to sleep at night. Most dogs were settled by being left in a room/area within the home without human company—59.4% (1385/2332) and 53.6% (585/1091) of dogs aged 16 weeks and 12 months, respectively. Of owners who reported “other” responses in the free-text space, three and eight owners reported that their dog self-settled (Table 1).

There were 29.8% (693/2328) and 43.9% (475/1083) of owners who reported that their dog slept in more than one sleeping place (for example ‘in a kennel/crate’ and ‘on a dog bed’) in the 16 week and 12 month surveys, respectively. In the 16 week survey, the most commonly reported sleeping place was in a kennel/crate (66.6%, *n* = 1550/2328), but at 12 months, the most common sleeping place was on a dog bed (49.9%, *n* = 540/1083) (Table 1).

There were 24.4% (*n* = 526/2152) and 40.4% (441/1091) of dogs reported to be able to get close to people at night, and 86.7% (449/518) and 86.6% (382/441) of these dogs reportedly chose to be around people in the 16 week and 12 month surveys, respectively (Table 1).

The owners’ observations of additional sleep characteristics and behaviour at 12 months of age are summarised in Table 2. Owners were asked in what position their dog tended to sleep and could select multiple responses. Most commonly, two sleeping positions were selected (32.9%, *n* = 343/1042). The most common sleeping position reported was “stretched out on his/her side” (84.2%, *n* = 877), and the second most common was “in a ‘curled up’ position” (63.6%, *n* = 663). In the free-text responses, five owners reported that their dog slept with their head dangling (e.g., over the edge of a bed or chair), and four owners reported that their dog slept on their stomach with front and back paws stretched out (Table 2).

In dogs aged 12 months, the most commonly reported observation of behaviour during sleep was “has small twitching movements of his/her legs” (73.0%, 761/1042). In the free-text responses, owners reported that their dog: slept peacefully (*n* = 78), made noises (e.g., barks, squeaks or whimpers) (*n* = 15), snored quietly (*n* = 8), twitched facial muscles or ears (*n* = 5), and wagged tail (*n* = 2) (Table 2). Of the 137 dogs that were reported to have “often snored very loudly while asleep”, 97 dogs were purebreds and summary statistics are provided in Table 3 for the seven most common purebred dogs (*n* = 51).

## 4. Discussion

This study sought to address a gap in existing research by describing sleep duration, sleep characteristics and behaviours for a large sample of dogs living in domestic environments at two timepoints (16 weeks and 12 months of age). The study identified that 16-week-old puppies slept longer compared to 12-month-old dogs. This increase was primarily driven by an increase in time reported as asleep during the day. Other differences in sleep behaviours and characteristics were also identified.

The median time owners reported that their dogs slept during the day was 3.5 h in the 16 week survey and 3.0 h in the 12 month survey; the difference was statistically significant. This estimate of duration of daytime sleep is slightly longer than the 3.2 h which was previously reported for dogs aged 1.5 years [21]. The mean average nighttime sleep was 7.0 h and 7.3 h in the 16 week survey and the 12 month survey, respectively. This difference was also statistically significant. In two previous studies, dogs aged over 1.5 years spent 60–80% of a 12 h nighttime period asleep (i.e., 7.2–9.6 h) [3,21], with younger dogs (aged 1.5–4.5 years) spending less time asleep than older dogs (aged 11–14 years) [21]. However, our results need to be interpreted (and compared to previous studies) cautiously, as neither “daytime” nor “nighttime” sleep periods were defined and both terms were left to the owner to interpret. It is plausible, for example, that owners thought of “daytime” as a longer period than between 8 am and 8 pm (period used in previous studies). Interpretations of these periods may have also been different in the summer when “daytime” could have been perceived as longer than in winter. These factors could have led to an observation of a longer daytime sleep than reported in the previous research.

Within our study, the mean total hours owners reported that their dog slept in a 24 h period was 11.2 h for 16-week-old puppies and 10.8 h for 12-month-old dogs. This is slightly longer than a previous study that reported 10.1 h to be the average sleep time within a 24 h period [19]. The differences may have arisen from different definitions of sleep periods or differences in methods for measuring sleep used in the other studies. Overall, owners within the ‘Generation Pup’ cohort reported that their dogs spent more time asleep during the daytime and a similar time asleep during the nighttime compared to previous studies. Previous research shows that adolescence is linked with substantial changes in sleep architecture and duration [31,32]. Although in this study, sleep architecture was not explored, the additional time spent asleep could be a function of the comparatively younger age of dogs in our study.

Within the ‘Generation Pup’ cohort, dogs aged 16 weeks spent significantly more time asleep during the day and in total, while dogs aged 12 months spent more time asleep at night. This was contrary to previous research which showed that as dogs age they spend longer asleep during a 24 h period [15,21]. Changes in sleep architecture and duration after learning have been previously observed [7,9]. The longer sleep duration at the 16 week timepoint supports the idea that young puppies were adapting to a new home environment and learning new skills, as the owners’ observations were made relatively soon after the puppies joined their new household. Future research within the ‘Generation Pup’ study will compare sleep behaviour of home-bred puppies to puppies that experienced a change in home environment to explore this idea further.

A limitation of this study is that owners were not directly observing dogs’ sleep and may not have been able to accurately identify when their dog was asleep (as opposed to resting) during the daytime or may not have been able to report precisely how long their dog slept overnight. The perception of how long a dog slept may have been influenced by the owner’s own sleeping behaviour, in particular at the 12 month timepoint when more dogs were reported to have access to people at night than at the 16 week timepoint. In this exploratory study, we did not collect data on the owner’s sleeping patterns, which may have affected a dog’s sleep duration and other behaviours. In addition, the nighttime sleep duration data provided by owners who did not sleep in the same room as their dogs should be treated with caution, due to potential for inaccuracies in reporting. As the ‘Generation Pup’ cohort increases in size, further investigations will explore the owner-perceived sleep duration for dogs that sleep in the same room as owners compared to those who do not, how this varies over time, and how this associated with health and behaviour outcomes.

At both timepoints, owners most commonly reported that their dog was settled to sleep at night by being left in a room/area within the home without human company. Compared to the 16 week survey, at 12 months of age, a considerably lower percentage of dogs were reported to sleep in a kennel/crate, and the percentages of dogs sleeping on a dog bed, a human bed, the floor or other furniture were higher. The 12 month survey also saw an increase, from 29.8% to 37.5%, between the two timepoints, in dogs being settled to sleep in a bedroom with human company. Proportionally more dogs were also reported to have access to humans at night at the later versus earlier timepoint (40.4% versus 24.4%). Previous research reported that 29% of puppies had access to people at nighttime during the first week of being in the new owners’ house, which is similar to our findings at 16 weeks of age [33]. The changes reported here could be a consequence of fewer dogs sleeping in a kennel/crate at 12 months of age. It is plausible that the sleep location of 16-week-old puppies may have been restricted for reasons such as the puppy might not have been completely housetrained. In the 16 week survey, several owners noted in the free-text box that their puppy needed access to puppy training/toileting pads overnight. Owners could have also started off with the intention for their dog to sleep in a particular place, but over time relaxed the original intention. Previous research showed that “nuisance behaviour” (such as vocalising, door scratching and seeking contact with the owner) that woke up the owners during the night was common in newly rehomed puppies, and in response, owners often allowed dogs access to people during the nighttime, albeit with a varied effect on dog behaviour [34]. An increase in dog’s access to owners at night could reflect similar motivations.

At 16 weeks and 12 months, respectively, 86.7% and 86.8% of dogs that had access to people overnight chose to be around them. Previous research into human–animal co-sleeping has primarily focused on the impact on the owner, and mixed effects have been reported. One survey-based study reported longer periods of time required to fall asleep and increased tiredness on waking [34]. In a study that used actigraphy measures (non-invasive monitoring rest/activity cycles using a sensor), dog movement predicted human movement [17]. Conversely, another actigraphy study reported good sleep efficiency in individuals who shared their bedroom (but not their bed) with a dog(s) [35]. Several studies have also suggested that some social benefits and/or increased sense of security could be obtained from co-sleeping [35,36]. It is plausible that dogs’ sleep is also influenced by sleeping near people. Further research is required here, and into the potential effects of preventing a dog from having access to people over night.

In the 12 month survey, the most common sleeping behaviour owners observed was “small twitching movements” of their dog’s legs. These reported observations may be limited by the owners’ observations skills as it is plausible that some behaviours were more difficult to observe/interpret (e.g., occasional disruptions in breathing or dog dreaming a lot). Further, 137 dogs were reported by their owners to “often snored very loudly”, and eight dogs reportedly snored “quietly” in the free-text box, thus owners may have had different interpretations of what “often” and “very loudly” meant. In addition, due to campaigns raising awareness of Brachycephalic Obstructive Airway Syndrome, owners of breeds known to commonly suffer from this condition may have been reluctant to report that their dog often snored or loudly snored due to social desirability bias. However, Pugs and French Bulldogs are two well-known flat-faced breeds, and 100% and 66.7% of dogs of those breeds, respectively, were reported to often snore loudly. The number of dogs of each of these two breeds is low in the cohort, and to provide the opportunity for more meaningful analysis, more dogs of these breeds need to be recruited.

Like other longitudinal cohort studies, the ‘Generation Pup’ cohort may suffer from a self-selection bias and may also be less able to attract and retain participants from lower socioeconomic backgrounds [37]. These two factors may have implications on how representative the dogs recruited for this project are with respect to breed and investment in dog health and care.

## 5. Conclusions

To use sleep behaviour as an indicator of welfare of dogs, normal age-specific sleep patterns and behaviours need to be better understood. Although owner reports may suffer from recall bias, they have good ecological validity and have practical value, as they are easier to access than other sleep measurements. The sleep behaviours described by owners within this large sample offer an indication of normal age-specific sleep patterns in young dogs and provide a baseline against which other populations of dogs at these age points can be compared. Future work within the ‘Generation Pup’ study will seek to describe the variation in health and behavioural issues in this cohort so that it is possible to assess how representative the findings are of normal healthy dogs. In addition, due to the longitudinal nature of the ‘Generation Pup’ study, the same dogs can be studied over time. It is, therefore, hoped that the results presented here will be utilized as a baseline for future ‘Generation Pup’ research. In further research, multivariable analysis will be used to explore factors important in sleep behaviour and duration, and how sleep behaviour and duration are associated with dog training, behaviour and welfare indicators of dogs as well as with health outcomes.

## Figures and Tables

**Table 1:** Summary of sleep characteristics, as reported by owners participating in the ‘Generation Pup’ study when their dogs were 16 weeks (*n* = 2332) and 12 months of age (*n* = 1091).

	16-Week-Old Dogs N (%)	12-Month-Old Dogs N (%)
**How the dog was settled to sleep at night** *(Owners could select one response only)*		
By leaving him/her in a room/area without human company	1385 (59.4)	585 (53.6)
By having him/her in a bedroom with human company	695 (29.8)	409 (37.5)
By sleeping in another room in the house with him/her (e.g., sleeping in the living room)	117 (5.0)	56 (5.1)
By waiting for him/her to be asleep before going to bed in a different room	99 (4.2)	14 (1.3)
By leaving him/her in an outbuilding (e.g., kennel, stable)	16 (0.7)	15 (1.4)
Free-text ‘other’ responses ^1^	20 (0.9)	12 (1.1)
**What the dog slept on/in the last seven days** *(Owners could select multiple responses)*		
In a kennel/crate	1550 (66.6)	393 (36.3)
On a dog bed	903 (38.8)	540 (49.9)
On the floor	312 (13.4)	298 (27.5)
On a person’s bed	310 (13.3)	291 (26.9)
On a chair/other furniture	95 (4.1) ^2^	184 (17.0) ^3^
**Whether the dog can get close to people if he/she chose at night**		
Yes	526 (24.4)	441 ( 40.4)
No	1626 (75.6) ^4^	650 (59.6)
**Of dogs that can get close to people, whether the dog choose to be close to people**		
Yes	449 (86.7)	382 ( 86.6)
No	69 (13.3) ^5^	59 (13.4)

^1^ Three and eight owners reported that their dog settled by him/herself in the 16 week and 12 month free-text ‘other’ responses, respectively. Missing data: ^2^ for 4 dogs, ^3^ for 8 dogs, ^4^ for 180 dogs, and ^5^ for 8 dogs.

**Table 2:** Summary of owner-observed sleeping behaviours, as reported by 1091 owners participating in the ‘Generation Pup’ study when their dogs were 12 months of age. (Owners could select multiple responses for both questions shown.)

	12 Month Survey Response N (%)
**The position the dog tended to sleep in**	
Stretched out on his/her side	877 ( 84.2)
In a ‘curled up’ position	663 (63.6)
On his/her back	419 (40.2)
With his/her head propped up (for sample on the side of his/her bed)	411 (39.4)
With a toy/object in his/her mouth	39 (3.7)
I don’t know, as I don’t see him/her sleep much/at all	6 (0.6)
Free-text ‘other’ responses	29 (2.8) ^1,2^
**Observations by the owner when the dog was asleep**	
Has small twitching movements of his/her legs	761 ( 73.0)
Dreams a lot	391 (37.5)
Looks as if he/she is chasing something	312 (29.9)
Often snores very loudly when he/she is asleep	137 (13.1)
Wakes up frequently/has disturbed sleep	89 (8.5)
Is restless	19 (1.8)
Stops breathing sometimes	5 (0.5)
I don’t know, as I don’t see him/her sleep much/at all	77 (7.4)
Free-text ‘other’ responses	150 (14.4) ^2,3^

^1^ In the free-text ‘other’ responses, five owners reported that their dog slept with their head dangling (e.g., over the edge of a bed or chair), and four owners reported that their dog slept on their stomach with front and back paws stretched out. ^2^ Missing data for 49 dogs. ^3^ In the free-text ‘other’ responses, owners reported that their dog: slept peacefully (*n* = 78), made noises (e.g., barks, squeaks or whimpers) (*n* = 15), snored quietly (*n* = 8), twitched facial muscles or ears (n = 5), and wagged tail (*n* = 2).

**Table 3:** Summary of the seven most common breeds reported by their owners to “often snore very loudly” when asleep at 12 months of age (*n* = 51).

	Reported to “Often Snore very Loudly”		
Breed of Dog	Yes N	No N	Percentage That “Often Snore very Loudly” (%)
Labrador Retriever	23	81	21.1
English Cocker Spaniel	7	19	26.9
Border Collie	5	58	7.9
Cavalier King Charles Spaniel	4	12	25.0
Clumber Spaniel	4	0	100.0
French Bulldog	4	2	66.7
Pug	4	0	100.0

## Appendix A

Sleep-related questions from the ‘Generation Pup’ 16 week survey and 12 month survey.

**My puppy sleeps during the nighttime for a total of approximately….**				
	*Please write number of hours below*	*or*	*Tick the box below*	
	Mininum of_________hour sMaximum of_________hours		I’m not sure how long	☐
**My puppy sleeps during the day-time for a total of approximately….**				
	*Please write number of hours below*	*or*	*Tick the box below*	
	Mininum of_________hours Maximum of_________hours		I’m not sure how long	☐
**At the moment, my puppy is settled to sleep at night...** *(tick one box)*				
	By having him/her in a bedroom with human company			☐
	By waiting for him/her to be asleep before going to bed in a different room			☐
	By leaving him/her in a room/area without human company			☐
	By sleeping in another room in the house with him/her (for example sleeping in the living room)			☐
	By leaving him/her in an outbuilding (for example kennel, stable)			☐
	Other (please specify)			☐
**At night my puppy sleeps...** *(tick all that apply)*				
	On the floor			☐
	On furniture (other than a bed)			☐
	On a human bed			☐
	In a kennel/crate			☐
	On a dog bed			☐
	I do not know			☐
	Other (please specify)			☐
**We would like to know if your puppy can get close to people if he/she chooses during the night...** *(tick one box)*				
	Yes (for example doors are open)			☐
	No			☐
*Follow on question, if you ticked ‘Yes’ to the above question, please answer this question.*				
**Does your puppy usually choose to be close to people at night?** *(tick one box)*				
Yes				☐
No				☐

Additional sleep-related questions from the ‘Generation Pup’ 12 month survey.

**When my dog is asleep I have noticed he/she…** *(tick all that apply)*	
Dreams a lot	☐
Often snores very loudly when he/she is asleep	☐
Looks as if he/she is chasing something	☐
Has small twitching movements of his/her legs	☐
Stops breathing sometimes	☐
Is restless	☐
Wakes up frequently/has disturbed sleep	☐
I don’t know, as I don’t see him/her asleep much/at all	☐
Other (please specify)	☐
**I notice that my dog tends to sleep…** *(tick all that apply)*	
In a ‘curled up’ position	☐
Stretched out on his/her side	☐
On his/her back	☐
With a toy/object in his/her mouth	☐
With his/her head propped up (for example on the side of his/her bed)	☐
I don’t know, as I don’t see him/her asleep much/at all	☐
Other (please specify)	☐
